# Metodologías para la priorización en investigación en salud: una revisión sistemática de la literatura

**DOI:** 10.26633/RPSP.2017.122

**Published:** 2017-11-30

**Authors:** Juan Camilo Fuentes, Lorena Andrea Cañón, Ángela Viviana Pérez, Carlos E. Pinzón, Angélica María Pérez, Paola Astrid Avellaneda, Álvaro Enrique Morales, y Jorge Enrique Fernández

**Affiliations:** 1 Instituto de Evaluación Tecnológica en Salud Instituto de Evaluación Tecnológica en Salud Bogotá Colombia Instituto de Evaluación Tecnológica en Salud, Bogotá, Colombia.; 2 Instituto Nacional de Salud Instituto Nacional de Salud Bogotá Colombia Instituto Nacional de Salud, Bogotá, Colombia.; 3 Ministerio del Trabajo Ministerio del Trabajo Bogotá Colombia Ministerio del Trabajo, Bogotá, Colombia.

**Keywords:** Investigación, prioridades en salud, métodos, revisión, Research, health priorities, methods, review, Pesquisa, prioridades em saúde, métodos, revisão

## Abstract

**Objetivo.:**

Identificar elementos metodológicos clave para la priorización en investigación en salud, a partir de las metodologías reportadas en la literatura científica.

**Métodos.:**

*Se realizó una búsqueda sistemática en Medline, Embase, LILACS, y fuentes complementarias de literatura gris. Se utilizaron las palabras clave:* research, methods *y* health priorities, *en combinación con términos libres. Dos revisores independientes, de acuerdo con criterios previamente definidos, seleccionaron revisiones de la literatura o documentos metodológicos que presentaran metodologías para priorización en investigación en salud. Se extrajeron las principales características de las metodologías reportadas y se identificaron elementos comunes.*

**Resultados.:**

Se incluyeron siete revisiones y cinco documentos metodológicos, que reportaron cuatro metodologías estructuradas específicas y múltiples aproximaciones metodológicas que combinan elementos diversos. En general, estas metodologías integran la perspectiva de actores clave con información objetiva, mediante la aplicación de técnicas estandarizadas de participación, para establecer un ranking de prioridades, con base en criterios previamente definidos. Se identificaron elementos metodológicos comunes relacionados con pasos del proceso, mecanismos de participación, criterios para priorizar y análisis de resultados.

**Conclusión.:**

*La priorización en investigación en salud requiere el empleo de una metodología definida* a priori, *que debe contener como mínimo cuatro elementos clave: pasos claros del proceso, criterios para priorizar, técnicas formales de participación y métodos de análisis de resultados. Estos elementos deben ajustarse a las condiciones y necesidades del contexto de aplicación.*

## Abstract (es)

**Objective.:**

To identify key methodological elements for prioritization in health research, based on the methodologies reported in the scientific literature.

**Methods.:**

A systematic search was conducted in Medline, Embase, LILACS, and complementary sources of gray literature. Keywords research, methods and health priorities were used in combination with free terms. Two independent reviewers, according to previously defined criteria, selected literature reviews or methodological documents that presented methodologies for prioritization in health research. The main characteristics of the reported methodologies were extracted and common elements were identified.

**Results.:**

Seven revisions and five methodological documents were included, reporting four specific structured methodologies and multiple methodological approaches combining diverse elements. In general, these methodologies integrate the perspective of key stakeholders with objective information, through the application of standardized participation techniques, to establish a ranking of priorities based on previously defined criteria. Common methodological elements related to process steps, participation mechanisms, criteria for prioritizing and analysis of results were identified.

**Conclusion.:**

The prioritization in health research requires the use of a methodology defined a priori, which must contain at least four key elements: clear steps of the process, criteria to prioritize, formal techniques of participation and methods for analysis of results. These elements should be tailored to the conditions and needs of the application context.

## RESUMO

**Objetivo.:**

Identificar os principais aspectos metodológicos para a priorização em pesquisa em saúde segundo metodologias descritas na literatura científica.

**Métodos.:**

Foi realizada uma busca sistemática nas bases de dados MEDLINE, Embase, LILACS e fontes complementares da literatura cinzenta. Foram usadas as palavras-chave *research, methods* e *health priorities*, em combinação com termos livres. Dois revisores independentes selecionaram, com base em critérios predefinidos, estudos de revisão da literatura ou textos metodológicos que descreviam metodologias para a priorização em pesquisa em saúde. Foram extraídas as principais características das metodologias descritas e identificados os aspectos comuns.

**Resultados.:**

Foram incluídos sete estudos de revisão e cinco textos metodológicos, que descreviam quatro metodologias estruturadas específicas e vários enfoques metodológicos que combinavam diversos aspectos. As metodologias em geral integram a perspectiva de atores-chave com informação objetiva, com a aplicação de técnicas padronizadas de participação, a fim de determinar a ordem de prioridade segundo critérios predefinidos. Foram identificados aspectos metodológicos comuns relacionados às etapas do processo, mecanismos de participação, critérios para priorização e análise de resultados.

**Conclusão:**

A priorização em pesquisa em saúde requer o uso de uma metodologia definida *a priori*, que no mínimo deve englobar quatro aspectos principais: etapas distintas do processo, critérios para priorização, técnicas formais de participação e métodos de análise de resultados. Estes aspectos devem ser adaptados às condições e necessidades do contexto de aplicação.

La investigación en salud es reconocida como una herramienta para mejorar la salud y la equidad en las poblaciones, así como un catalizador del desarrollo socioeconómico (1–4). Las necesidades de investigación en salud en un contexto específico y los potenciales beneficios de ésta son amplios y variados; no obstante, los recursos disponibles para su ejecución son limitados en cualquier escenario, particularmente en países de bajos y medianos ingresos (5).

Se estima que los recursos invertidos en investigación en salud a nivel global ascienden a US$ 130 mil millones anuales, con una tendencia continua al incremento en la década pasada. Sin embargo, en la actualidad los requerimientos de financiación exceden los recursos disponibles (5, 6), por lo que es esencial que la asignación de éstos responda más directamente a las necesidades de la comunidad que a los intereses de los investigadores o de los organismos financiadores, con el propósito de que los resultados de la investigación tengan el mayor impacto posible en la sociedad y la salud pública (7).

La asignación de recursos a la investigación en salud debe, en consecuencia, ser el resultado de una adecuada priorización, entendida como el proceso cuyo objetivo es determinar cuáles áreas del conocimiento producirán mayores beneficios a la sociedad, en respuesta a un esfuerzo investigativo incrementado que incluya colaboración, coordinación e inversión (8). Dicho proceso debe ser legítimo y justo, y llevado a cabo con una metodología racional, sistemática, explícita y transparente (5, 8, 9), lo cual es parte integral de cualquier proceso de gestión de la investigación (9).

La priorización en investigación en salud es un proceso complejo que implica un reto metodológico porque el número de ideas de investigación suele ser grande, los resultados son inherentemente inciertos, y el impacto de los mismos es difícil de predecir y medir (10), lo que ha incentivado la evolución de los métodos y la implementación de diferentes aproximaciones metodológicas durante las últimas tres décadas (7).

Algunos autores han resaltado la necesidad de mejorar el rigor metodológico de los ejercicios de priorización en investigación, particularmente en países de Latinoamérica. No obstante, no existe acuerdo acerca de cuál es la mejor aproximación metodológica, o cuáles son los elementos que deben ser tomados en cuenta para el diseño de la metodología de este tipo de ejercicios (11).

Se han publicado revisiones de la literatura orientadas a describir metodologías y resultados de procesos de priorización en investigación. Sin embargo, no han estado orientadas a presentar herramien-tas o elementos metodológicos concretos que orienten la elaboración de metodologías de priorización en procesos futuros. El objetivo de esta revisión sistemática fue identificar elementos metodológicos clave para la priorización en investigación en salud a partir de las metodologías reportadas en la literatura científica. En el marco de esta revisión, elementos metodológicos clave se entienden como aspectos de la metodología que son fundamentales y su identificación tiene el propósito de orientar a investigadores y tomadores de decisiones en el nivel nacional, local o institucional en el diseño de ejercicios de priorización en investigación en salud.

## MÉTODOS

Se llevó a cabo una revisión sistemática de la literatura con base en los lineamientos de la declaración Preferred Reporting Items for Systematic Reviews and Meta-Analyses (PRISMA) (12). La búsqueda de literatura se realizó en diciembre de 2015, sin restricción de fecha ni de idioma, en las bases de datos especializadas Pubmed, Embase y LILACS; y en las siguientes fuentes complementarias en búsqueda de literatura gris: motores de búsqueda genéricos (Google y Google Académico), técnica de bola de nieve, consulta a expertos y páginas web de organizaciones internacionales dedicadas al apoyo y fortalecimiento de sistemas nacionales de investigación, como la Organización Mundial de la Salud, la Organización Panamericana de la Salud, la Organización para la Cooperación y el Desarrollo Económicos y la Organización de las Naciones Unidas.

Se utilizó una estrategia de búsqueda genérica con términos MeSH, Emtree y lenguaje libre, la cual se adaptó para las diferentes fuentes de información (cuadro 1).

Luego de la eliminación de duplicados, dos revisores de manera independiente tamizaron las referencias resultantes, por revisión de título y resumen, y posteriormente confirmaron el cumplimiento de los criterios de elegibilidad mediante revisión de los textos completos de las referencias anteriormente seleccionadas.

Previamente se definieron los parámetros de elegibilidad utilizando como criterios de inclusión: 1) que el documento fuera una revisión de la literatura que reportara metodologías utilizadas en ejercicios de priorización en investigación en salud, o un artículo o documento metodológico enfocado en la priorización en investigación en salud. Se consideraron como artículos o documentos metodológicos, los estudios o documentos técnicos que pusieran a prueba de forma empírica, propusieran o describieran metodologías o elementos metodológicos; y 2) que el documento estuviera disponible en texto completo, en idioma inglés o español.

**CUADRO 1:** Estrategias de búsqueda para bases especializadas

Base de datos	Estrategia
PUBMED	((((((research [MeSH Terms]) OR research [Title/Abstract]) OR investigation [Title/Abstract])) AND ((methods [MeSH Major Topic]) OR method*[Title])) AND ((Health Priorities[MeSH Terms]) OR priori*[Title/Abstract])) AND priori*[Title]
EMBASE	#1'research priority'/exp OR 'research priority' 2,457 #2 prior*:ab,ti 721,730 #3 #1 OR #2 722,548 #4 'research'/exp 675,94 #5 'research':ti 219,184 #6 investigation:ti 99,599 #7 #4 OR #5 OR #6 888,333 #8 'methodology' 1,787,323 #9 method*:ti 452,819 #10#8 OR #9 2,117,327 #11 #3 AND #7 AND #10 AND prior*:ti 505
LILACS	(tw:(priorización)) AND (tw:(lineas de investigación)) OR (tw:(agenda de prioridades de investigación)) AND (tw:(metodologia)) OR (tw:(metodos))

***Fuente:*** Elaboración propia.

Se excluyeron 1) documentos correspondientes a una versión previa de un estudio incluido, y 2) documentos incluidos en una revisión de la literatura seleccionada, que no presentaran información adicional

Se incluyeron los documentos que cumplieron los criterios de elegibilidad, sin tener en cuenta su estado de publicación. Los desacuerdos entre revisores fueron solucionados mediante revisión conjunta y en caso de no lograr acuerdo, por juicio de un tercer revisor.

La calidad de los documentos incluidos no fue evaluada debido a que el objetivo de la revisión es descriptivo, y no implica un juicio crítico de la validez interna o externa de los resultados presentados.

Se extrajeron y describieron las principales características de las metodologías reportadas, según el tipo de documento (cuadro 2), y se identificaron elementos comunes, los cuales se propusieron como elementos metodológicos clave para la priorización en investigación en salud.

**CUADRO 2:** Información extraída de acuerdo al tipo de documento

Revisiones de la literatura	Documentos metodológicos
Título	Título
Autor/Año	Autor/Año
País	País
Número de estudios incluidos	NA
Nivel de los ejercicios descritos (local, nacional, regional, internacional, otro)	NA
Escenario de los ejercicios descritos (internacional, países de altos, medianos o bajos ingresos, otro)	NA
Metodologías descritas	NA
Pasos del proceso de determinación de prioridades	Pasos descritos del proceso de determinación de prioridades
Criterios para priorizar	Criterios para priorizar
Métodos de participación	Métodos de participación
Métodos de análisis y establecimiento de prioridades	Métodos de análisis y establecimiento de prioridades

***Fuente:*** Elaboración propia. **NA:** No aplica.

**FIGURA 1:**
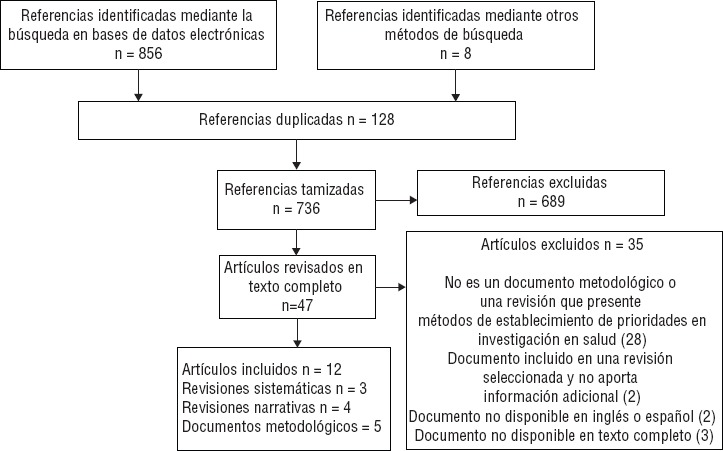
Diagrama de flujo PRISMA

***Fuente:*** Elaboración propia.

## RESULTADOS

### Búsqueda y selección

Se identificaron 736 referencias, de las cuales 47 fueron revisadas en texto completo. Doce documentos cumplieron los criterios de elegibilidad (5, 7–9, 11, 13–19), y se excluyeron 35: 28 por no corresponder a revisiones de la literatura que presentaran métodos para priorización en investigación en salud o a documentos metodológicos (10, 20–45), 2 por ser documentos incluidos en una revisión seleccionada y no aportar información adicional (46, 47), 2 por no estar disponibles en idioma inglés o español (48, 49), y 3 por no estar disponibles en texto completo (50–52). Los detalles del resultado del proceso de selección se presentan en la figura 1. El listado de los documentos excluidos y los motivos de exclusión están disponibles previa solicitud a los autores.

### Documentos incluidos

Se incluyeron 5 documentos metodológicos, 4 revisiones narrativas y 3 revisiones sistemáticas de la literatura (cuadro 3).

Los documentos metodológicos fueron publicados desde el año 2002 al 2013 en Canadá, Croacia, Egipto, Filipinas y el Reino Unido (5, 13, 16–18). Cuatro describieron metodologías estructuradas específicas mencionadas en las revisiones incluidas, pero proporcionaron información adicional a la presentada en éstas. Dichas metodologías fueron la *James Lind Alliance* (17), *Combined Approach Matrix* (CAM) (5), *Child Health Research Investments* (CHNRI) (13), y *European Network for Housing Research* (ENHR) (16). El quinto documento metodológico, “*Methodology for the development of a Canadian national EMS research agenda*”, describió los detalles de una metodología diseñada y aplicada en el desarrollo de la agenda nacional de investigación para los servicios médicos de emergencia en Canadá (18).

Las revisiones narrativas fueron publicadas entre 2006 y 2015 en Australia, Italia, Sudáfrica y Suiza, y describieron métodos para priorización en investigación en salud en el siglo XXI (14), en países de ingreso alto (8), en países de ingresos bajos y medianos (9), o en Seguridad y Salud en el Trabajo (15).

Las revisiones sistemáticas se publicaron entre los años 2013 y 2015, en Australia y Estados Unidos. Describieron los métodos utilizados para la priorización en investigación en salud en países de bajo y mediano ingreso (7), en Latinoamérica y el Caribe (11), y una, específicamente para investigación en enfermedades renales (19).

### Metodologías para la priorización

Las características de las metodologías reportadas en cada documento incluido son presentadas en detalle en el material suplementario. Ocho documentos describieron metodologías estructuradas específicas: CHNRI, *James Lind Alliance*, CAM y ENHR (5, 7, 9, 11, 13, 14, 16, 17). Estas metodologías presentan un marco estructurado y reproducible para la determinación de prioridades.

Los métodos CHNRI (13), *James Lind Alliance* (17) y ENHR (16), se basan en: a) conformación de un grupo adecuadamente seleccionado de actores clave; b) análisis del contexto; c) identificación de necesidades o propuestas de investigación (que se fundan frecuentemente en puntos de incertidumbre en el manejo clínico de una enfermedad); y d) valoración de éstas mediante diferentes técnicas de participación.

El CHNRI y el ENHR proponen la selección de acuerdo al contexto de criterios para priorizar, con base en los cuales se debe realizar la valoración de lasnecesidades o propuestas de investigación identificadas. La valoración se lleva a cabo asignando puntos a las propuestas o estimando subjetivamente la probabilidad de que cumplan los criterios establecidos para priorizar. La determinación de las prioridades se basa en el establecimiento de un ranking de propuestas de acuerdo con los puntos obtenidos o las probabilidades estimadas en la valoración de las mismas.

**CUADRO 3:** Características generales de los documentos incluidos

Autor	Año	Título	País	Tipo de documento
Cowan	2013	The James Lind Alliance Guidebook (17)	Reino Unido	Documento metodológico
Jensen	2011	Methodology for the development of a Canadian national EMS research agenda (18)	Canadá	Documento metodológico
Ghaffar	2009	Setting research priorities by applying the combined approach matrix (5)	Egipto	Documento metodológico
Rudan	2008	Setting Priorities in Global Child Health Research Investments: Guidelines for Implementation of CHNRI Method (13)	Croacia	Documento metodológico
Okello	2002	Un Manual para el Establecimiento de Prioridades de Investigación usando la estrategia de INES (ENHR) (16)	Filipinas	Documento metodológico
Yoshida	2015	Approaches, tools and methods used for setting priorities in health research in the 21^st^ century (14)	Suiza	Revisión narrativa
Bryant	2014	Health research priority setting in selected high income countries: a narrative review of methods used and recommendations for future practice (8)	Australia	Revisión narrativa
Tomlinson	2011	A review of selected research priority setting processes at national level in low and middle income countries: Towards fair and legitimate priority setting (9)	Sudáfrica	Revisión narrativa
Iavicoli	2006	Research priorities in occupational safety and health: a review (15)	Italia	Revisión narrativa
Tong	2015	Research Priority Setting in Kidney Disease: A Systematic Review (19)	Australia	Revisión sistemática
McGregor	2014	How are health research priorities set in low and middle-income countries? A systematic review of published reports (7)	Australia	Revisión sistemática
Reveiz	2013	Comparison of national health research priority-setting methods and characteristics in Latina America and the Caribbean, 2002–2012 (11)	Estados Unidos, México, Honduras, Suiza	Revisión sistemática

***Fuente:***Elaboración propia.

En contraste, el método CAM (5) se basa en la estimación objetiva en la medida de lo posible, de un conjunto establecido de variables que son analizadas por un grupo de actores clave, quienes deben identificar brechas de información y proponer proyectos de investigación que puedan solventarlas.

La mayoría de los estudios incluidos en las revisiones identificadas no reportaron el uso de ninguna de estas metodologías, y en su lugar describieron aproximaciones metodológicas variables, desarrolladas para cada caso particular, con elementos similares relacionados con: a) pasos del proceso; b) uso de criterios preestablecidos para priorizar; c) métodos de participación utilizados; d) métodos de análisis de resultados y establecimiento de prioridades.

### Pasos del proceso

Siete documentos reportaron los pasos de las metodologías que describieron (5, 8, 13, 15–18). Se observó variabilidad en el número, objetivos y métodos de los pasos reportados, pero en general comprendieron: a) adecuada selección de un grupo asesor, coordinador o decisor, compuesto por expertos en el tema o por actores clave; b) provisión a los miembros de estos grupos con información relevante para el proceso, relativa a los objetivos y métodos a utilizar, la evidencia pertinente disponible, proveniente de revisiones de la literatura o estadísticas de carga de la enfermedad, y estado del conocimiento, entre otros; c) generación e integración, mediante diferentes estrategias, de listas de propuestas de investigación; d) discusión y valoración de la importancia o prioridad de las propuestas de investigación mediante distintos métodos de participación, con componentes variables de objetividad y subjetividad; e) análisis de resultados y establecimiento de prioridades.

### Criterios para priorizar

Siete documentos (5, 7, 8, 11, 13, 15, 16) describieron criterios propuestos para valorar la importancia o la prioridad de las propuestas de investigación. Los criterios propuestos, a excepción de los establecidos en la metodología CAM (5), deben ser cuidadosamente seleccionados en función de las condiciones de cada ejercicio particular de priorización, y se pueden agrupar en las siguientes categorías: a) carga o frecuencia de la enfermedad/magnitud del problema; b) uso o retorno de los recursos invertidos; c) determinantes o factores de riesgo; d) viabilidad de la investigación o de la implementación de los resultados; e) impacto esperado de los resultados; f) aspectos legales; g) aspectos éticos; h) aceptabilidad/interés social o político; i) contribución potencial a la equidad; j) contribución potencial al conocimiento; k) contribución potencial al desarrollo económico o social; y l) contribución potencial al sistema de salud.

La selección de los criterios depende del juicio técnico de los investigadores o tomadores de decisiones, las características del contexto, el grado de objetividad esperado en la evaluación de los mismos, y las características del área del conocimiento de la investigación.

### Métodos de participación

Todos los documentos incluidos reportaron momentos de participación de actores clave en al menos un paso metodológico del proceso. Diez documentos (7, 8, 11, 13–19) reportaron alternativas metodológicas para los procesos participativos. Los métodos de participación más frecuentemente reportados fueron: a) Delphi; b) Delphi modificado; c) grupo nominal; d) panel/consenso de expertos; e) grupo focal; f) encuesta; g) taller.

La participación de actores clave fue descrita como un elemento fundamental, incluso en aquellas metodologías basadas en información objetiva con la CAM (5), a partir del reconocimiento de que es poco probable que se disponga de datos objeti-vos de toda la información requerida, y de que es importante el análisis de dicha información por expertos para sustentar la toma de decisiones. El grado y los momentos de participación de actores fue variable, pero en la mayoría de los casos se reportó el uso de técnicas estandarizadas.

### Métodos de análisis y establecimiento de prioridades

Ocho documentos presentaron lineamientos para el análisis de los resultados del proceso y el establecimiento de prioridades (5, 8, 9, 11, 13, 16–18), los cuales variaron dependiendo de la metodología propuesta. En general, se identificaron tres enfoques para el análisis de la información y la determinación de prioridades.

El primer enfoque se basa en el análisis cualicuantitativo de una combinación variable de datos objetivos y subjetivos disponibles, para concluir en la selección de los proyectos o temas de investigación que pueden llenar las brechas del conocimiento identificadas, tal como se propone en la metodología CAM (5). El uso de este tipo de análisis fue el menos reportado.

El segundo enfoque fue la determinación de las prioridades de investigación por diferentes técnicas de consenso partiendo de la información obtenida a lo largo del proceso, como describió Jensen en el año 2011 (18).

El último enfoque fue el más frecuentemente reportado y consistió en el establecimiento de un ranking de las propuestas de investigación con base en puntos asignados a cada una de ellas mediante distintas estrategias que se pueden resumir en: a) aplicación de métodos de análisis de grupo nominal, en el que se establecieron listados de propuestas en orden creciente o decreciente de prioridad (53–55); b) asignación de puntos mediante la utilización de escalas con niveles desde 0–1 hasta 1–9, que pueden ser aplicadas por cada criterio o de forma general a cada propuesta de investigación (16) para obtener un puntaje total, como resultado de la suma o multiplicación de los puntos asignados por criterio (13, 16), o de la media o la mediana de los puntos por propuesta de investigación (15, 16); y c) número de veces que cada propuesta fue mencionada en el proceso (15).

## DISCUSIÓN

Se identificaron y describieron métodos de priorización en investigación en salud implementados o propuestos en distintos continentes y para países de bajos, medianos y altos ingresos en las dos últimas décadas, lo cual permite mostrar un panorama amplio de las alternativas metodológicas disponibles para diversos escenarios.

Los resultados de las revisiones mostraron, de forma consistente, que la mayoría de los ejercicios de determinación de prioridades en investigación utilizaron metodologías desarrolladas para cada caso particular, y se identificaron elementos comunes que conectaron diferentes aproximaciones metodológicas descritas, que se proponen en esta revisión como elementos clave para el diseño metodológico de un ejercicio de determinación de prioridades en investigación en salud: a) determinar pasos claros del proceso; b) definir criterios para priorizar; c) generar espacios de participación con actores clave; d) determinar la meto-dología de análisis y de establecimiento de prioridades.

Estos elementos son coherentes con los criterios propuestos por Viergever y cols. para orientar el diseño y la evaluación de la calidad de los ejercicios de priorización en investigación en salud (56). Estos criterios comprenden: a) evaluación del contexto; b) uso de una aproximación metodológica exhaustiva; c) inclusión adecuada y representativa de los actores clave; d) recopilación de información; e) planeación de la imple-mentación; f) selección de criterios relevantes para priorizar; g) evaluación del proceso y de las prioridades establecidas; h) reporte claro y transparente del proceso.

Los elementos claves propuestos también son coherentes con los cuatro requerimientos planteados por Bryant (8) para un adecuado proceso de establecimiento de prioridades, que consisten en: a) un grupo asesor multidisciplinario debe supervisar y guiar el proceso de establecimiento de prioridades; b) una amplia representación de las partes interesadas es crítica; c) criterios para priorizar y objetivos claramente definidos deben guiar la generación de prioridades; d) el impacto de los procesos de es-tablecimiento de prioridades debe ser evaluado.

Los resultados de esta revisión fueron consistentes con los de autores que han mostrado que no existe un estándar de referencia para la priorización en investigación en salud (8, 11, 14).

Se han utilizado dos tipos de aproximaciones metodológicas: metodologías estructuradas específicas y aproximaciones metodológicas diseñadas para ejercicios puntuales que combinan diversos elementos. No obstante, no existe consenso respecto a cuál alternativa tiene mayor validez (56), por lo que se requiere en el futuro evaluar si un solo método debe ser adaptado para la mayoría de los contextos, o si el desarrollo de aproximaciones que combinen elementos y fortalezas de varias alternativas metodológicas reporta mayores beneficios (14).

Algunos autores plantean que los métodos estructurados específicos proporcionan mayor solidez, reproducibilidad y transparencia a los procesos de determinación de prioridades (14), en tanto que otros argumentan que la diversidad de contextos hace que la aplicación de una metodología única no sea posible, y de hecho sea inapropiado (8, 56). En cualquiera de los casos, hay acuerdo en que las metodologías deben establecer procesos y herramientas ajustadas a cada escenario de aplicación.

A pesar de que no fueron identificados como elementos comunes en las aproximaciones metodológicas descritas, varios autores plantearon la necesidad de evaluar el proceso y sus resultados, y de construir un plan para la ejecución de las prioridades establecidas (7, 8, 56), y se recomienda que se considere su inclusión en procesos exhaustivos de determi-nación de prioridades en investigación.

La principal fortaleza de este estudio fue integrar sistemáticamente los resultados de revisiones de la literatura previamente realizadas, y complementarlas mediante la actualización de la búsqueda de artículos primarios o documentos técnicos metodológicos, sin tener un límite en el escenario de aplicación o en el área de interés del conocimiento en salud. Asimismo, expone de manera exhaustiva las alternativas y herramientas metodológicas que han sido reportadas en la literatura para proponer elementos metodológicos que no deben ser pasados por alto y que constituyen una guía para el diseño de cualquier ejercicio de priorización en investigación en salud, lo cual no había sido realizado en las revisiones previamente publicadas.

El alcance de la revisión no permite la determinación de las variables que están asociadas a la utilización de las diferentes alternativas metodológicas descritas, ni la comparación de las ventajas y desventajas de éstas, en diferentes escenarios de aplicación.

Una limitación de esta revisión fue la exclusión de documentos no disponibles en idioma inglés o español, lo cual excluyó dos artículos potencialmente relevantes.

Debido a la naturaleza no cuantitativa de la información objeto de la revisión, no se realizó una evaluación formal de la probabilidad de sesgo de publicación, que se puede producir por la no identificación de metodologías propuestas por instituciones de poco reconocimiento o no publicadas en revistas indexadas. Sin embargo, se implementaron estrategias para minimizar esta probabilidad, tales como la búsqueda en fuentes complementarias de información y la inclusión de documentos sin tener en cuenta su estado de publicación.

En conclusión, la priorización de investigación en salud requiere una metodología reproducible y transparente definida *a priori*, bien sea una metodología estructurada específica o una aproximación metodológica compuesta de elementos metodológicos diversos, que contenga como mínimo cuatro elementos clave: pasos claros del proceso, criterios para priorizar, técnicas formales de participación y métodos de análisis de resultados. Estos elementos deben ajustarse al campo del conocimiento, a las condiciones y a las necesidades del contexto de aplicación. Se sugiere incluir en los ejercicios de determinación de prioridades en investigación la evaluación del proceso, la construcción de un plan de ejecución y el seguimiento al desarrollo de la investigación priorizada y al impacto de los resultados.

### Financiación.

Esta revisión fue realizada con fondos del Ministerio del Trabajo, en el marco del convenio 355/2014, entre el Ministerio del Trabajo, el Instituto Nacional de Salud y el Instituto de Evaluación Tecnológica en Salud, todas instituciones de Colombia; que tuvo como objetivo establecer las líneas de investigación en salud laboral prioritarias para Colombia

### Declaración.

Las opiniones expresadas en este manuscrito son responsabilidad del autor y no reflejan necesariamente los criterios ni la política de la *RPSP/PAJPH* y/o de la OPS.
